# Theoretical Investigation on Indirect Tensile Strength of Concrete with Rectangular Cross-Section under Locally Distributed Load

**DOI:** 10.3390/ma16247671

**Published:** 2023-12-15

**Authors:** Ziran Wang, Jialin Xu, Linhao Sun, Jinchao Yue, Quansheng Zang

**Affiliations:** 1 School of Water Conservancy and Transportation, Zhengzhou University, Zhengzhou 450001, China; wzr823868@163.com (Z.W.); jaelyn522@163.com (J.X.); yuejc@zzu.edu.cn (J.Y.); 2 Zhengzhou Public Utility Investment and Development Group Co., Ltd., Zhengzhou 450000, China; sunlinhao2023@163.com

**Keywords:** rectangular, concrete, indirect tensile strength, triangular solution, loading width, length to height ratio

## Abstract

The indirect tensile test plays a crucial role in experimental investigations of brittle material properties. In this study, a mechanical analysis model of the rectangular test block is established based on the theory of elastic mechanics for the characteristics of the indirect tensile test. The theoretical solution of the triangular series is derived for the rectangular test block under the locally distributed load. The finite element simulation results and splitting test results were compared with the theoretical results. The results of the study verify the accuracy of the theoretical solutions. Based on the proposed analytical solution, the effects of loading width and length-to-height ratio (*h*/*l*) of local loading on the measured tensile strength of test block are discussed. The results demonstrate that the tensile strength of the test block increases as the loading width expands, and the rate of growth in the recorded tensile strength gradually stabilizes. The variation in loading width affects the location of crack initiation points during the concrete test block splitting tests. When the loading width exceeds 6% of the side length of test block, the cracking point is positioned at the center of test block, ensuring the effectiveness of the splitting test. As the length-to-height ratio of the test block increases, there is a general upward trend in the measured tensile strength. When *h*/*l* < 0.6, the measured tensile strength initially increases before decreasing. However, when *h*/*l* > 0.6, the measured tensile strength consistently increases, with the rate of increase gradually diminishing until it stabilizes. The length-to-height ratio also significantly influences the location of the cracking point in the test block. As the length-to-height ratio increases, the cracking point initially shifts from around the center to the central point and then further from the center toward the edge. To ensure that the location of the crack initiation point is in the center of the specimen and that the tensile strength is close to the measured result, the length to height ratio can be chosen at around 0.85.

## 1. Introduction

Tensile strength in brittle materials such as rocks and concrete is typically determined using the direct tensile method or the indirect tensile method [1,2,3]. Shorter test cycles and more accurate results are achieved with the indirect tensile method compared to the direct tensile method [4,5,6,7,8,9]. Indirect tensile strength is measured through two traditional methods, namely the Brazilian test and the cube cleavage test. The Brazilian test involves a cylindrical splitting test, which ensures uniform tensile stress on the splitting surface and yields reliable results [10,11]. Nevertheless, the collection and fabrication of the test block, as well as the application of the load, are more demanding. The cube cleavage test offers convenience in both specimen fabrication and loading. Its formula for indirect tensile strength is adopted from the Brazilian disc splitting tensile strength solution [12,13]. This may lead to a discrepancy between the experimental results and the actual situation.

Researchers have made many efforts to minimize this error. Some researchers use machine learning methods to predict the splitting strength [14,15,16,17]. Van Cauwelaert [18] provided an approximate analytical solution for the splitting tensile strength of rectangular test blocks by modifying the equation using a two-dimensional planar body stress function of infinite length in the horizontal direction. Based on the test data of the cylinder transverse and the test data of cube, Chai et al. [19] established a relationship equation between the cylinder splitting strength (*f*_2_) and the splitting strength (*f*_1_) of the cube. Huang et al., Luan et al. and Ince et al. [20,21,22] also established different prediction models for cube splitting strength based on the experimental results. But the reliability of the prediction model based on the experimental results will be affected by the experimental environment or other factors. Therefore, in this paper, based on the plane theory of elastic mechanics, the theoretical study of rectangular cross-section specimen splitting test problem is carried out to determine the theoretical solution of rectangular cross-section specimen splitting test triangular series iteration.

Currently, a comprehensive and precise theoretical analytical solution for indirect tensile strength testing of rectangular specimens is not available. In this study, the analytical solution of the stress component at any point in the stressed rectangular specimen is derived based on the theory of elastic mechanics analysis for the characteristics of the indirect tensile test of rectangular specimens. And numerical simulation tests were carried out using the finite element software Abaqus 2014, and the theoretical results, numerical simulation results and existing actual experimental results were compared and analyzed. Based on the theoretical analytical formula, the tensile strength of brittle material was analyzed theoretically, and the effects of the width of the load action and the length-to-height ratio of the specimen on the tensile strength were investigated. The theoretical analytical solution for the indirect tensile strength of rectangular specimens is presented in this paper, which bridges a current gap in the theory of indirect tensile strength testing using square specimens at present.

## 2. Trigonometric Series Solution of the Indirect Tensile Strength of Rectangular Test Blocks

### 2.1. Brief Description of the Theoretical Solution Method for Indirect Tensile Strength of Rectangular Specimen

The splitting test of the rectangular section specimen under distributed load is similar to the splitting test of the circular section specimen. The splitting test of the rectangular section specimen is generally solved by triangular series, and the stress function selected in the process of solving has a greater impact on the accuracy of the calculation results and the complexity of the operation.

The locally distributed load acting on the interface between the upper and lower sides of the rectangular test block is converted into a continuous load function by means of a triangular series expansion. On the basis of assuming the stress function in the right-angled coordinate system, the expression of the stress function and the analytical solution of the stress component at any point in the specimen are obtained according to the semi-inverse solution method of the plane stress problem.

### 2.2. Theoretical Solutions for Rectangular Test Blocks under Locally Distributed Loads

The locally distributed load acting on the interface between the upper and lower sides of the rectangular test block is converted into a continuous load function via triangular level expansion. On the basis of the assumed stress function in the right-angle coordinate system, the expression of the stress function and the analytical solution of the stress component at any point within the test block are obtained using the semi-inverse solution method of the plane stress problem [23,24,25].

**Figure 1:**
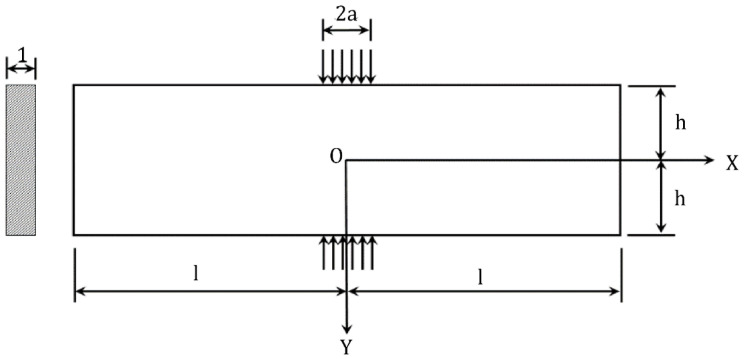
Mechanical calculation model of indirect tensile strength of rectangular specimen block.

The rectangular test block is subjected to a uniform load of size *q* and width 2*a* on the upper and lower faces. The test block has a side length of 2*l*, a height of 2*h*, and a unit thickness of 1. The coordinate system is established at the center of the square test block, and the mechanical model is displayed in Figure 1. The stress function *U* [10] is of the following form:

(1) $$U={(K}_{1}cos\lambda x+K_{2}sin\lambda x)(Acosh\lambda y+Bsinh\lambda y+Cycosh\lambda y+Dycosh\lambda y)$$

where *K*_1_*, K*_2_, *A*, *B*, *C*, *D* and *λ* are constants.

The boundary conditions can be written as

(2) $$x=\pm l\;\sigma_{x}=0$$

(3) $$y=\pm h\;\sigma_{y}=-f(x)$$

(4) $$x=\pm l,\;y=\pm h\;\tau_{xy}=0$$

The stress components in this case are

(5) $$\sigma_{x}=\frac{\partial^{2}U}{\partial y^{2}}$$

(6) $$\sigma_{y}=\frac{\partial^{2}U}{\partial x^{2}}$$

(7) $$\tau_{y}=-\frac{\partial^{2}U}{\partial x\partial y}$$

Considering the expression of stress component $\sigma_{x}$ and the boundary condition described in Equation (2), i.e., at x=±l, $\sigma_{x}=\partial^{2}U/\partial y^{2}=0$, we find

(8) $$K_{2}=0$$

(9) $$\lambda_{m}=\frac{\left(2m+1\right)\pi }{2l}\;(m=0,\;1,\;2,\;3,\;\ldots )$$

$\lambda_{m}$ is a parameter related to the number of terms calculated in the calculation process. It was mentioned above that *K*_1_ is a constant factor. Substituting Equations (8) and (9) into the stress function Equation (1), and making $K_{1}=1$, the following equation can be obtained after a summation operation

(10) $$U=\sum_{m=0}^{\infty }\cos\;\lambda_{m}x\left(A_{m}\;\cos h\;\lambda_{m}y+B_{m}\;\sin h\;\lambda_{m}y+C_{m}y\cos h\;\lambda_{m}y+D_{m}y\cos h\;\lambda_{m}y\right)$$

The following stress components are obtained by taking the partial derivative of Equation (10) from the expression of the stress function for the stress components.

(11) $$\sigma_{x}=\sum_{m=0}^{\infty }{\lambda_{m}}^{2}[A_{m}cosh\lambda_{m}y+B_{m}sinh\lambda_{m}y+C_{m}(ycosh\lambda_{m}y+\frac{2}{\lambda_{m}}sinh\lambda_{m}y)\;+D_{m}(ysinh\lambda_{m}y+\frac{2}{\lambda_{m}}cosh\lambda_{m}y)]cos\lambda_{m}x$$

(12) $$\sigma_{y}=-\sum_{m=0}^{\infty }{\lambda_{m}}^{2}(A_{m}cosh\lambda_{m}y+B_{m}sinh\lambda_{m}y+C_{m}ycosh\lambda_{m}y+D_{m}ysinh\lambda_{m}y)cos\lambda_{m}x$$

(13) $$\tau_{xy}=\sum_{m=0}^{\infty }{\lambda_{m}}^{2}[A_{m}sinh\lambda_{m}y+B_{m}cosh\lambda_{m}y+C_{m}(ysinh\lambda_{m}y+\frac{2}{\lambda_{m}}cosh\lambda_{m}y)\;+D_{m}(ycosh\lambda_{m}y+\frac{2}{\lambda_{m}}sinh\lambda_{m}y)]sin\lambda_{m}x$$

The loads applied on the upper and lower boundaries of the rectangular domain can be described in the following function form

(14) $$f\left(x\right)=\left\{\begin{array}{ll} q & \left(-a\leq x\leq a\right)\\ 0 & \left(x<-a,\;x>a\right) \end{array}\right.$$

The boundary conditions, as determined by the stress function, exhibit continuity. Consequently, the load function on the boundary is expanded into a continuous trigonometric series form. Furthermore, the load function on the boundary is an even function, and the expansion of its trigonometric series can be expressed as:

(15) $$f(x)=\sum_{n=0}^{n=\infty }a_{n}\cos\frac{\left(2n+1\right)\pi x}{2l}$$

$a_{n}$ are then

(16) $$a_{n}=\frac{1}{l}\int_{-a}^{a}f(x)\cos\frac{\left(2n+1\right)\pi x}{2l}dx\;\left(n=0,\;1,\;2,\;3,\;\ldots \right)$$

The integration operation on Equation (16) gives the result of

(17) $$a_{n}=\frac{4q}{\left(2n+1\right)\pi }\sin\frac{(2n+1)\pi a}{2l}$$

Substituting Equation (17) into Equation (15), the triangular series expansion of the load can further be expressed as

(18) $$f(x)=\sum_{n=0}^{\infty }\frac{4q}{\left(2n+1\right)\pi }\sin\frac{\left(2n+1\right)\pi a}{2l}\cos\frac{\left(2n+1\right)\pi x}{2l}$$

From the boundary condition Equation (3), it can be known that $\sigma_{y}$ holds the same value when y=±h; this implies $\sigma_{y}$ is an even function. Thanks to the properties of even functions, it can be concluded that

(19) $$B_{m}=C_{m}=0$$

Considering Equation (19), and substituting Equation (18) as well as Equation (12) into Equation (3) in the case of y=±h, yields

(20) $$\sum_{m=0}^{\infty }{\lambda_{m}}^{2}(A_{m}\;\cos h\;\lambda_{m}y+D_{m}y\sin h\;\lambda_{m}y)\cos\lambda_{m}x=\sum_{n=0}^{\infty }\frac{4q}{\left(2n+1\right)\pi }\sin\frac{\left(2n+1\right)\pi a}{2l}\cos\frac{\left(2n+1\right)\pi x}{2l}$$

Comparing the two side coefficients, it can be seen that when m=n, the two sides are equal.

(21) $$A_{m}\cos h\lambda_{m}y+D_{m}y\sin h\lambda_{m}y=E_{m}$$

Bringing Equation (12) to the left side of the Equation (13) produces

(22) $$E_{m}=\frac{4q}{\left(2m+1\right)\pi }\sin\frac{\left(2m+1\right)\pi a}{2l}\frac{1}{{\lambda_{m}}^{2}}$$

When *y = ±h*, ${\tau_{x}}_{y}=0$

(23) $$A_{m}\sin h\lambda_{m}y+D_{m}(y\cos h\lambda_{m}y+\frac{2}{\lambda_{m}}\sin h\lambda_{m}y)=0$$

Equations (21) and (23) are solved by the combination of

(24) $$A_{m}=2E_{m}\frac{\sin h\;\lambda_{m}h+\lambda_{m}h\sin h\;\lambda_{m}h}{2\lambda_{m}h+\sin h2\;\lambda_{m}h}$$

(25) $$D_{m}=-2E_{m}\frac{\sin h\;\lambda_{m}h}{2\lambda_{m}h+\sin h\;2\lambda_{m}h}$$

Replacing *A_m_* and *D_m_* into the stress component expression, the expression of the stress component at any point in the rectangular test block under locally distributed load is:

(26) $$\sigma_{x}=\sum_{m=0}^{\infty }\lambda_{m}[A_{m}\lambda_{m}cosh\lambda_{m}y+D_{m}(y\lambda_{m}sinh\lambda_{m}y+2cosh\lambda_{m}y)]cos\lambda_{m}x$$

(27) $$\sigma_{y}=-\sum_{m=0}^{\infty }{\lambda_{m}}^{2}(A_{m}cosh\lambda_{m}y+D_{m}ysinh\lambda_{m}y)cos\lambda_{m}x$$

(28) $$\tau_{xy}=\sum_{m=0}^{\infty }\lambda_{m}[A_{m}\lambda_{m}sinh\lambda_{m}y+D_{m}(y\lambda_{m}cosh\lambda_{m}y+2sinh\lambda_{m}y)]sin\lambda_{m}x$$

## 3. Verification of Theoretical Results

### 3.1. Comparison of Theoretical Solutions and Finite Element Simulation Tests

To verify the accuracy of the theoretical analytical solution of the requested stress components, the finite element software (ABAQUS) is used to simulate the splitting test of rectangular test blocks under local loading. The theoretical results are compared and analyzed with the numerical simulation results.

**Figure 2:**
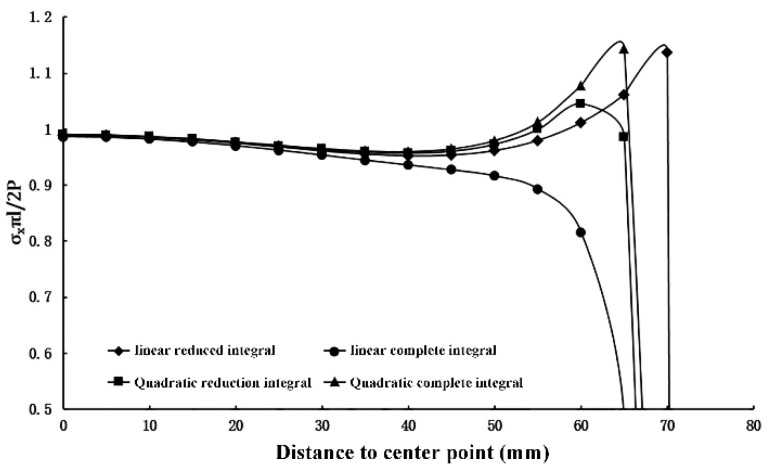
Influence of finite element cell type on calculation results.

The length (2*l*) and height (2*h*) of the rectangular model are 150 mm. The width of the load action (2*a*) is 1/15 of the side length, and the local loading is 100 MPa. The elastic modulus of the selected specimen is 3.0 × 10^4^ MPa, and Poisson’s ratio is 0.2. To assure accuracy, a quadrilateral grid is employed, and it is honed to 0.1 mm × 0.1 mm. The entire numerical model consists of 2,250,000 elements and 2,253,001 nodes. The cross-section type is solid and homogeneous. Parameter settings were established in the concrete damage plasticity model as follows: the expansion angle is 30°, the eccentricity is 0.1, the ratio of biaxial ultimate compressive strength to uniaxial ultimate compressive strength is 1.16, the constant stress ratio is 0.6667, while the cohesion coefficient is 0.0005, the damage factor is 0.9, and the other parameters are set to default values. The selection of the unit type is determined by comparing square specimens under concentrated force, as shown in Figure 2.

**Figure 3:**
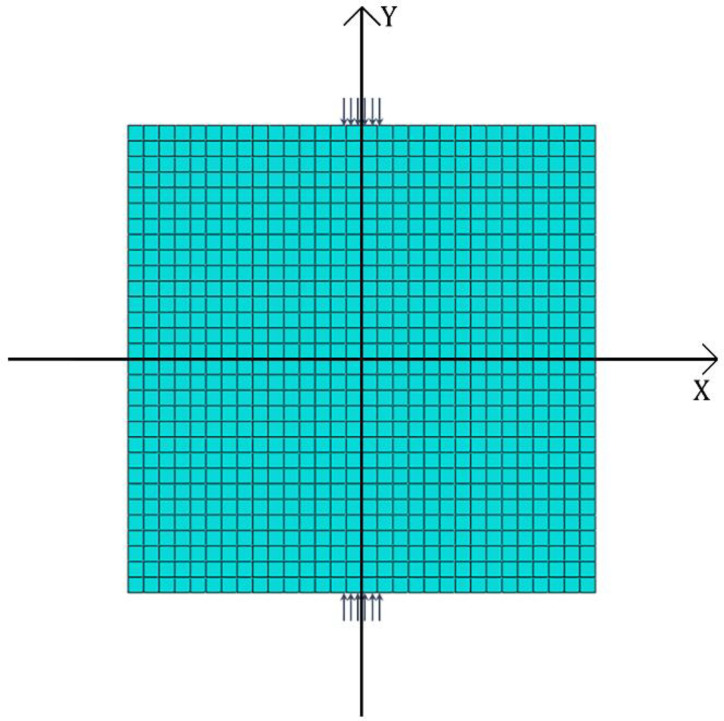
Finite element model.

The results obtained from the calculations of the four cell types are basically the same in the region near the center of the specimen, but there is a large variation in the range that produces stress concentrations near the loading point. The linear reduced integral has a large variation in the calculated results near the loading point of the specimen, and appears to be distorted. The trend of the calculated results of linear full integral, quadratic reduced integral and quadratic full integral remains consistent with the increase in the calculation accuracy. The true value of the calculation results gradually tends to the result of using the quadratic complete integral unit. By comparison, the accuracy of the test results can be guaranteed by adopting the quadratic complete integral unit. A quadratic fully integrated plane stress cell (CPS8) is used. The schematic diagram of the finite element is illustrated in Figure 3. 

**Figure 4:**
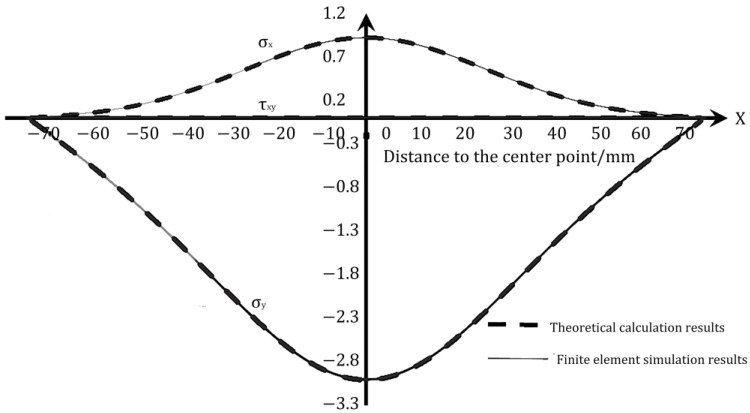
Comparison of theoretical results and simulation results of stress components on OX.

**Figure 5:**
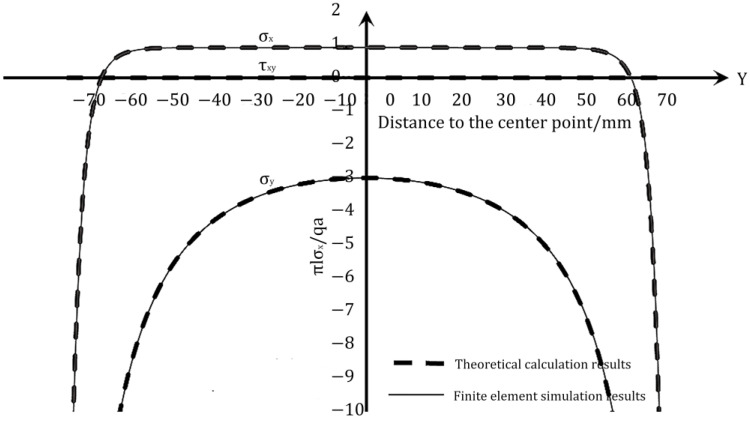
Comparison of theoretical results and simulation results of stress components on OY.

To compare the numerical simulation results with the theoretical results, the zero point of the coordinate axis in the center of the test block is identified and the values of stress components on each axis are compared. The results of the analysis are displayed in Figure 4 and Figure 5.

As can be seen in Figure 4 and Figure 5, the numerical simulation results and the theoretical results are symmetrical at the center for this size and load. Both the numerical simulation results and the theoretical results are consistent with the actual situation. The overall change trend of the numerical simulation results and the theoretical results in the figure is the same, and the theoretical results of the horizontal stress components on both axes match well with the numerical simulation results. Thus, it can be verified that the theoretical results are accurate and reasonable.

**Figure 6:**
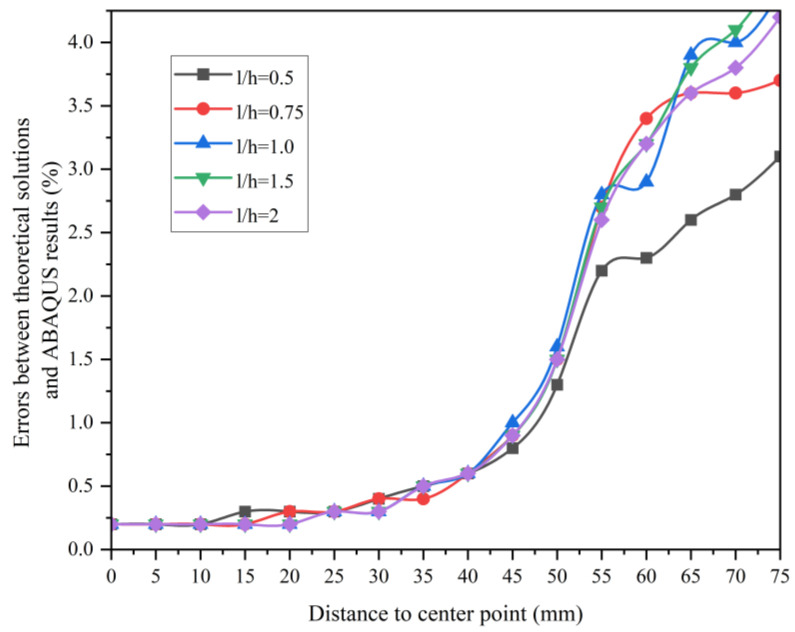
Errors between theoretical solutions and ABAQUS calculations for the splitting test of rectangular section specimens.

The errors between the theoretical solutions and the ABAQUS calculations for the splitting tests of the rectangular section specimens are listed in Figure 6. The correctness of the theoretical results is verified by finite element simulation. So theoretical analysis of the tensile strength of rectangular test blocks can be carried out using the theoretical analytical solution. The influence of the load action width and test block size on the measured tensile strength can be discussed.

### 3.2. Comparison between Theoretical Solutions and Experimental Results

**Figure 7:**
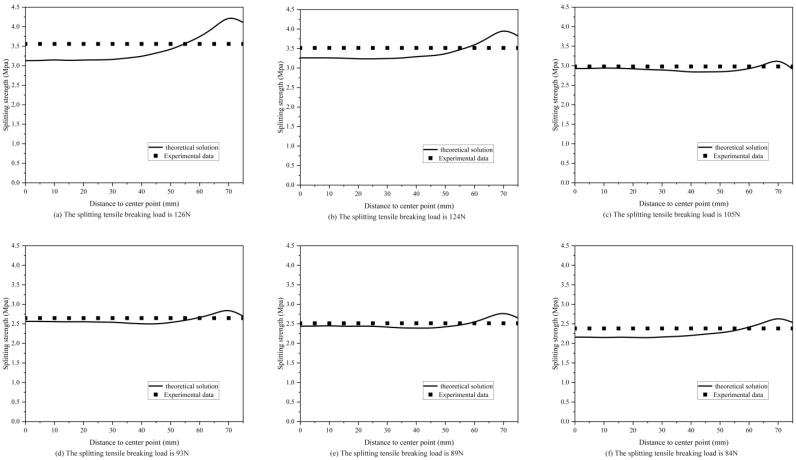
Splitting strength ratio of theoretical solutions to experimental results.

The rectangular section splitting test and the circular section splitting test are important components of the splitting test. The two forms of experimental methods have certain commonalities. The experimental results of cubic specimens are directly processed using the theoretical results of circular cross-section specimens. Chai et al. [19] conducted a large number of cube splitting tests. The measured splitting tensile strength fts is calculated using the following formula.

(29) $$f_{ts}=\frac{2F}{\pi A}=0.637\frac{F}{A}$$

where: *f_ts_* is the splitting tensile strength of the concrete cube (MPa); *F* is the ultimate load (N); *A* is the splitting surface area (mm^2^). The comparison results of the splitting strength are illustrated in Figure 7. Overall, the theoretical values correlate well with the measured data. From the figure, it can be observed that the ratio near the midpoint of the specimen is smaller and the ratio near the loading point is larger. This may be due to the fact that the measured results were calculated by approximating the rectangular cross section to a circular cross section, resulting in small values.

## 4. Theoretical Analysis of Tensile Strength of Rectangular Test Blocks under Locally Distributed Load

### 4.1. Effect of Local Loading Width on Tensile Strength

Research has demonstrated the significant impact of mat bar width on tensile strength. Rocco et al. [26] conducted experiments using 150 mm cubes and round steel matting bars of varying diameters. The choice of an appropriate loading width during testing ensures the validity of the splitting test and minimizes its influence on the calculation of splitting strength. The findings indicate that as the diameter of the mat bar increased, the measured tensile strength also increased [27]. As per the results obtained by Tang [28], the variation in the effect of pad width on specimens of different sizes can be eliminated when the pad ratio (the ratio of pad width to specimen side dimension) remains constant. Considering the influence of pad size on tensile strength, numerous researchers have made modifications to the equation specified in the code [29,30].

**Figure 8:**
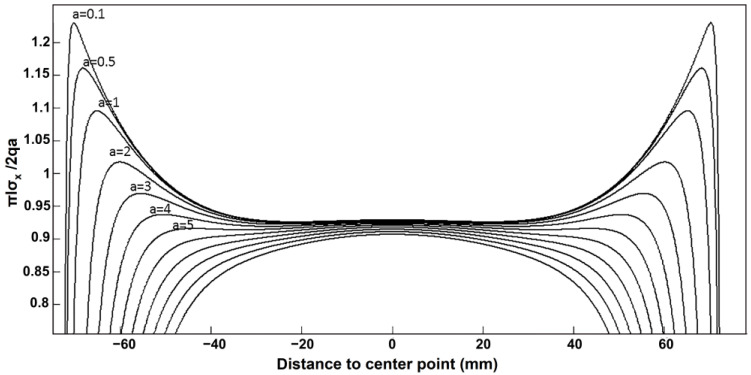
Variation curve of stress component $\sigma_{x}$ with loading width.

**Figure 9:**
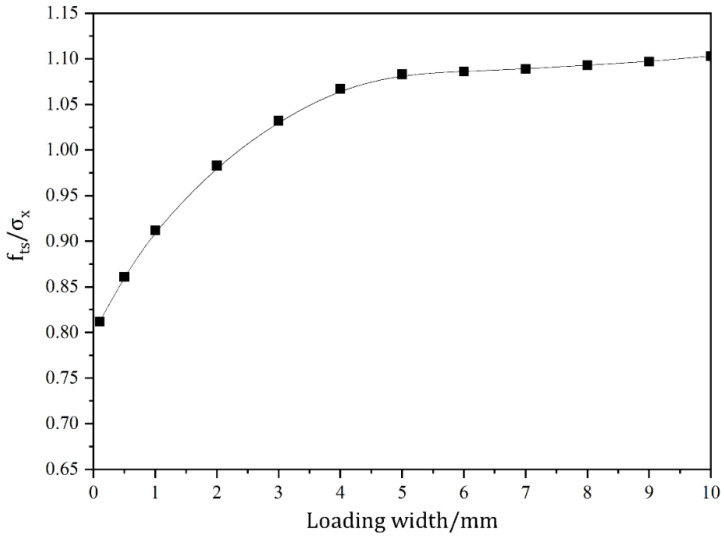
Variation curve of $f_{ts}/\sigma_{x}$ with loading width.

The rectangular side length (2*l*) and height (2*h*) are both assumed to be 150mm for the sake of conducting a theoretical study of the impact of load action width on the tensile strength of test blocks in this article. As indicated in Table 1, the values of the loading width (*a*) and the related values of tensile strength were computed. As the width of the loading action increases, the value of the distributed load decreases to ensure that the combined force remains constant. The variation of $\sigma_{x}$ against the distance to center point is displayed in Figure 8, followed by the variation curve of $f_{ts}/\sigma_{x}$, as exhibited in Figure 9.

As can be observed in Figure 8, with the increase in loading width, the maximum value of stress component $\sigma_{x}$ reveals a decreasing trend. The tensile strength of the specimen increases with the increase in the loading action width. Figure 9 exhibits that the stress concentration near the loading is obvious when the loading width is less than 6% of the side length of the specimen. In this case, the test block does not crack from the center point. When the loading width is too small, the test block will cause damage near the loading point due to stress concentration, resulting in indirect tensile strength test failure. The loading width will have a large impact on the location of the cracking point. When the loaded width is less than 6% of the length of the side, the cracking point is on both sides of the center. As the loading width increases, it gradually moves to the center. When the loading width is larger, the cracking point is located at the center of the specimen. With the increase in the loading width, the measured tensile strength presents an overall increasing trend. The rate of increase gradually stabilizes with the increase in loading width.

### 4.2. Effect of Length-to-Height Ratio of Rectangular Test Blocks on Tensile Strength

When considering the influence of size effect on the tensile strength, the analysis was divided into two cases with fixed aspect ratios as well as variable aspect ratios. It was indicated that in the case of fixed aspect ratios, the tensile strength decreased with increasing size [31,32,33,34]. This result can only be analyzed through experimental tests, while the effect of varying the length–height ratio on the measured tensile strength can be determined via theoretical analysis.

The stress distribution along the loading axis at the center of the specimen was calculated using the triangular step iterative solution of the splitting test for rectangular section specimens and the ABAQUS finite element numerical simulation, respectively. The length to height ratio of the rectangular section specimen was taken to be 0.5, 0.75, 1.0, 1.25, 1.5, 1.75 and 2.0. The height of the rectangular section specimen (2*h*) was 150 mm and the finite element calculation parameters were the same as those chosen in the above paper. It was assumed that the combined force of the applied distributed forces was 1000 N and the loading width was 0.2 [l]. The parameters of the computational model are expressed in Table 2.

**Figure 10:**
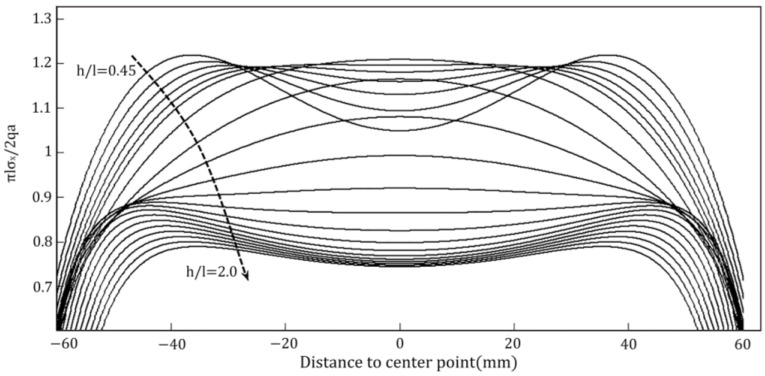
Variation of stress component *σ_x_* versus length-to-height ratio.

The effect of the length-to-height ratio on the internal stress concentration phenomenon of the specimen is relatively obvious. As evident from Figure 10, the length-to-height ratio significantly influenced the location of the crack initiation point in the specimen. With an increase in the aspect ratio, the cracking point initially shifted from both sides towards the center, and then reversed from the center back towards sides. The cracking point was situated in the center of the specimen when the aspect ratio fell within in the range of 0.6 to 1.0. Therefore, the rectangular cross-section specimen splitting test should be coordinated with the length and height ratio and loading width, to ensure the effectiveness of the splitting test, and accurately calculate the splitting strength value.

**Figure 11:**
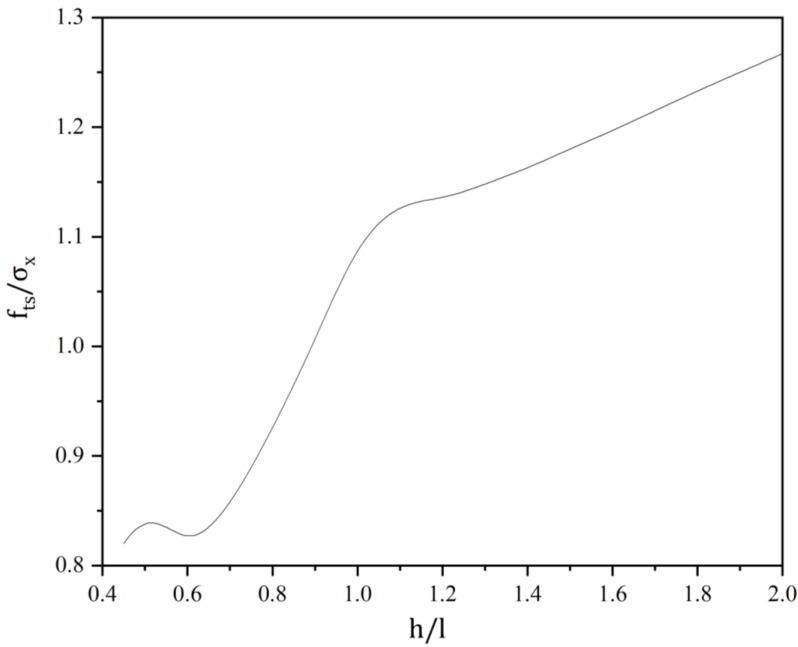
Variation in the tensile strength against the length-to-height ratio.

From the calculated results demonstrated in Figure 11, it is observed that the trend of tensile strength of rectangular specimens with varying length-to-height ratios is rather complicated. The tensile strength increases with an increase in the length-to-height ratio. When *h*/*l* < 0.6, the tensile strength initially rises before declining. Conversely, when *h*/*l* exceeds 0.6, tensile strength exhibits an increasing trend. Moreover, the rate of increase is more significant in the range of 0.6 < *h*/*l* < 1.1 compared to when *h*/*l* is greater than 1.1. 

From the above analysis, when rectangular blocks are used for splitting tests, the influence of the block length-to-height ratio on the measured results should be fully considered. To ensure that the location of the cracking point is in the center of the test block and the tensile strength is close to the measured results, the length-to-height ratio is chosen to be around 0.85.

Table 3 lists the comparison of errors between analytical and finite element solutions on stress distribution in specimens under different length-to-height ratios, in which *d* means the distance to the center point. It can be observed from Table 3 that the error between the finite element and theoretical results through ABAQUS is smaller. The effect of the loading width on the splitting test illustrates that the theoretical results are closer to the finite element calculation results when the stress concentration phenomenon at both ends of the specimen is weak. The bedding ratio of 0.2 used in the finite element calculations is slightly more inaccurate in the area of stress concentration at both ends relative to the center of the specimen. However, the results are more accurate for concentrated forces. Therefore, it is reasonable and accurate to analyze the effect of specimen length-to-height ratio on the splitting strength of specimens by means of the triangular step iteration theory of splitting tests for rectangular cross-section specimens.

## 5. Conclusions

In this paper, the analytical solution of the triangular series of indirect tensile strength of rectangular brittle materials specimens is proposed by using the theory of elastic mechanics analysis. The accuracy of the theoretical formulation is verified by comparing the analysis with the finite element simulation results. On the basis of this theoretical result, the effect of locally distributed load loading width and length-to-height ratio on the measured tensile strength is analyzed and the following conclusions are obtained:

- The measured tensile strength of the specimens increases with the increase in the load action width. When the width of load action is less than 6% of the side length of the added specimen, the stress concentration near the loading point is obvious and the measured tensile strength value is small. If the width of load action is too small, it will cause stress concentration damage near the loading point and make the splitting tensile strength test invalid.
- The width of the load action has a greater influence on the location of the cracking point. With the increase in the width of the load action, the cracking point gradually moves from both sides to the center. The measured tensile strength presents an increasing trend with the increase in the loading width, and the rate of increase will gradually stabilize.
- The measured tensile strength increases with the increase in the aspect ratio. When *h*/*l* < 0.6, the measured tensile strength increases first and then decreases; when *h*/*l* > 0.6, the measured tensile strength manifests an increasing trend, and the increase rate is greater when 0.6 < *h*/*l* < 1.1 than when *h*/*l* > 1.1.
- The length-to-height ratio has a greater influence on the location of the crack initiation point of the specimen. With the increase in the length-to-height ratio, the location of the crack initiation point first moves from both sides to the center point, and then moves from the center point to both sides.

## Figures and Tables

**Table 1:** Calculation results of load action width and tensile strength.

*a*/mm	0.1	0.5	1	2	3	4	5	6	7	8	9	10
*f_ts_*/*σ_x_*	0.812	0.861	0.912	0.983	1.032	1.067	1.083	1.086	1.089	1.093	1.097	1.103

**Table 2:** Calculated parameters for the effect of length to height ratio on stress distribution.

*h*/*l*	*h* (mm)	*l* (mm)	Loading Width (mm)	Distributed Load Values (MPa)
0.50	150.00	75.00	15.00	66.67
0.75	150.00	112.50	22.50	44.44
1.00	150.00	150.00	30.00	33.33
1.25	150.00	187.50	37.50	26.67
1.50	150.00	225.00	45.00	22.22
1.75	150.00	262.50	52.50	19.05
2.00	150.00	300.00	60.00	16.67

**Table 3:** Comparison of errors between analytical and finite element solutions on stress distribution in specimens under different length-to-height ratio.

*d* (mm)	*h*/*l* = 0.5	*h*/*l* = 0.75	*h*/*l* = 1	*h*/*l* = 1.25	*h*/*l* = 1.5	*h*/*l* = 1.75	*h*/*l* = 2
0	0.068%	0.066%	0.072%	0.050%	0.006%	0.001%	0.021%
5	0.069%	0.065%	0.072%	0.050%	0.006%	0.002%	0.021%
10	0.071%	0.064%	0.072%	0.049%	0.005%	0.002%	0.021%
15	0.074%	0.062%	0.071%	0.049%	0.003%	0.003%	0.022%
20	0.077%	0.058%	0.070%	0.049%	0.000%	0.005%	0.023%
25	0.078%	0.051%	0.069%	0.049%	0.003%	0.006%	0.025%
30	0.077%	0.041%	0.069%	0.050%	0.008%	0.009%	0.026%
35	0.072%	0.026%	0.070%	0.053%	0.013%	0.012%	0.028%
40	0.060%	0.003%	0.075%	0.058%	0.021%	0.016%	0.028%
45	0.036%	0.033%	0.087%	0.067%	0.033%	0.023%	0.026%
50	0.010%	0.093%	0.115%	0.085%	0.068%	0.053%	0.010%
55	0.100%	0.240%	0.246%	0.973%	0.141%	0.026%	0.044%
60	0.330%	3.924%	0.036%	0.067%	0.011%	0.005%	0.019%
65	2.984%	0.128%	0.097%	0.061%	0.024%	0.058%	0.087%
70	0.066%	0.000%	0.005%	0.152%	0.345%	0.511%	0.564%
75	3.996%	4.048%	4.233%	4.532%	4.864%	5.207%	5.567%

## Data Availability

Data are contained within the article.
